# Impact of virtual monoenergetic levels on coronary plaque volume components using photon-counting computed tomography

**DOI:** 10.1007/s00330-023-09876-7

**Published:** 2023-07-24

**Authors:** Borbála Vattay, Bálint Szilveszter, Melinda Boussoussou, Milán Vecsey-Nagy, Andrew Lin, Gábor Konkoly, Anikó Kubovje, Florian Schwarz, Béla Merkely, Pál Maurovich-Horvat, Michelle C. Williams, Damini Dey, Márton Kolossváry

**Affiliations:** 1 MTA-SE “Lendület” Cardiovascular Imaging Research Group, Semmelweis University Heart and Vascular Center, Városmajor Street 68., 1122 Budapest, Hungary; 2 Biomedical Imaging Research Institute, Cedars-Sinai Medical Center, 116 N Robertson Blvd, Suite 400, CA 90048 Los Angeles, USA; 3 Semmelweis University Medical Imaging Center, Korányi Sándor Street 2., 1082 Budapest, Hungary; 4 Clinic for Diagnostic and Interventional Radiology and Neuroradiology, University Hospital Augsburg, Stenglinstr. 2, 86156 Augsburg, Germany; 5 University of Edinburgh/British Heart Foundation Centre for Cardiovascular Science, 47 Little France Crescent, Edinburgh, EH16 4TJ UK; 6 Gottsegen National Cardiovascular Center, 29 Haller Utca, 1096 Budapest, Hungary; 7 Physiological Controls Research Center, University Research and Innovation Center, Óbuda University, Bécsi Út 96/B, 1034 Budapest, Hungary

**Keywords:** Coronary arteriosclerosis, Reproducibility of results, Atherosclerosis, CT angiography

## Abstract

**Objectives:**

Virtual monoenergetic images (VMIs) from photon-counting CT (PCCT) may change quantitative coronary plaque volumes. We aimed to assess how plaque component volumes change with respect to VMIs.

**Methods:**

Coronary CT angiography (CTA) images were acquired using a dual-source PCCT and VMIs were reconstructed between 40 and 180 keV in 10-keV increments. Polychromatic images at 120 kVp (T3D) were used as reference. Quantitative plaque analysis was performed on T3D images and segmentation masks were copied to VMI reconstructions. Calcified plaque (CP; > 350 Hounsfield units, HU), non-calcified plaque (NCP; 30 to 350 HU), and low-attenuation NCP (LAP; − 100 to 30 HU) volumes were calculated using fixed thresholds.

**Results:**

We analyzed 51 plaques from 51 patients (67% male, mean age 65 ± 12 years). Average attenuation and contrast-to-noise ratio (CNR) decreased significantly with increasing keV levels, with similar values observed between T3D and 70 keV images (299 ± 209 vs. 303 ± 225 HU, *p* = 0.15 for mean HU; 15.5 ± 3.7 vs. 15.8 ± 3.5, *p* = 0.32 for CNR). Mean NCP volume was comparable between T3D and 100–180-keV reconstructions. There was a monotonic decrease in mean CP volume, with a significant difference between all VMIs and T3D (*p* < 0.05). LAP volume increased with increasing keV levels and all VMIs showed a significant difference compared to T3D, except for 50 keV (28.0 ± 30.8 mm^3^ and 28.6 ± 30.1 mm^3^, respectively, *p* = 0.63).

**Conclusions:**

Estimated coronary plaque volumes significantly differ between VMIs. Normalization protocols are needed to have comparable results between future studies, especially for LAP volume which is currently defined using a fixed HU threshold.

**Clinical relevance statement:**

Different virtual monoenergetic images from photon-counting CT alter attenuation values and therefore corresponding plaque component volumes. New clinical standards and protocols are required to determine the optimal thresholds to derive plaque volumes from photon-counting CT.

**Key Points:**

• *Utilizing different VMI energy levels from photon-counting CT for the analysis of coronary artery plaques leads to substantial changes in attenuation values and corresponding plaque component volumes.*

• *Low-energy images (40–70 keV) improved contrast-to-noise ratio, however also increased image noise.*

• *Normalization protocols are needed to have comparable results between future studies, especially for low-attenuation plaque volume which is currently defined using a fixed HU threshold.*

**Supplementary Information:**

The online version contains supplementary material available at 10.1007/s00330-023-09876-7.

## Introduction

Coronary computed tomography angiography (CTA) allows characterization of atherosclerotic plaque in addition to luminal stenosis [1]. Quantifying coronary plaque burden and adverse plaque characteristics may improve cardiovascular risk prediction. Notably, low-attenuation non-calcified plaque (LAP) burden is an independent predictor of myocardial infarction [2].

Novel photon-counting CT (PCCT) is a promising technique for the assessment of coronary arteries with superior spatial and temporal resolution as compared with current-generation scanners [3]. Compared to conventional energy-integrating detectors, photon-counting detectors register the energy of each individual photon and directly convert x-ray photons to electrical signals without the need of reflecting septa, resulting in improved spatial resolution, noise reduction, and better soft tissue contrast [4]. It has previously been demonstrated on histological atherosclerotic plaque samples that different plaque components and vessel lumen can be accurately differentiated using spectral data from a PCCT system [5]. Furthermore, this allows for virtual monoenergetic images (VMIs) which may help evaluation of coronary CTA due to improvements in blooming artefact reduction and contrast-to-noise ratio (CNR) [6]. Also, PCCT allows for sharper delineation of structures such as calcifications, as it provides superior spatial resolution. Therefore, using different VMI reconstructions may improve coronary plaque detection due to changes in intraluminal contrast attenuation and CNR. However, VMIs also change the Hounsfield unit (HU) values of the voxels and therefore may impact plaque volume estimates which are often done using fixed HU thresholds.

Therefore, our aim was to assess how quantification of individual plaque components changes with respect to different monoenergetic levels obtained using PCCT.

## Materials and methods

### Study design and patient population

Consecutive patients referred for clinically indicated coronary CTA due to suspected or known coronary artery disease (CAD) were screened in our prospective, single-center study between April 2022 and June 2022. Inclusion criteria were (1) diagnostic image quality for quantitative plaque analysis and (2) discernible coronary lesion in at least one of the main coronary arteries. Exclusion criteria were (1) presence of stents or bypass grafts and (2) images with severe motion, breathing, beam-hardening, or misalignment artifacts.

The study was approved by the institutional ethical committee (IV/667–1/2022/EKU) and was performed in accordance with the Helsinki declaration. Written informed consent was obtained from all patients.

### Coronary CTA acquisition and reconstruction

ECG-triggered CTA scans of the heart were performed using a first-generation dual-source PCCT scanner (NAEOTOM Alpha, Siemens Healthineers). Coronary CTA imaging was obtained according to the guidelines of the Society of Cardiovascular Computed Tomography [7]. Scan parameters for all patients were as follows: tube voltage = 120 kVp, automatic tube current modulation with image quality level (IQ-level) = 80, detector configuration = 144 mm × 0.4 mm, rotation time = 0.25 s. Intravenous beta blocker was administered if heart rate (HR) was > 65 beats/minute before examination. All patients received 0.8 mg of sublingual nitroglycerine before CTA scanning if systolic blood pressure was > 100 mmHg. High-pitch helical (TurboFlash) scan mode was used if HR was regular and below 70/min, sequential scan mode was applied in case of regular HR > 70 beats/min, and helical scan mode was used if HR was irregular. Images were acquired in diastole (65–85% of the R-R interval) or systole (200–400 ms) depending on the HR (< or > 75 beats/minute). A four-phasic contrast injection protocol was used with 70–80 mL contrast agent at a flow rate of 4.5–5.0 mL/s [8].

**Fig. 1:**
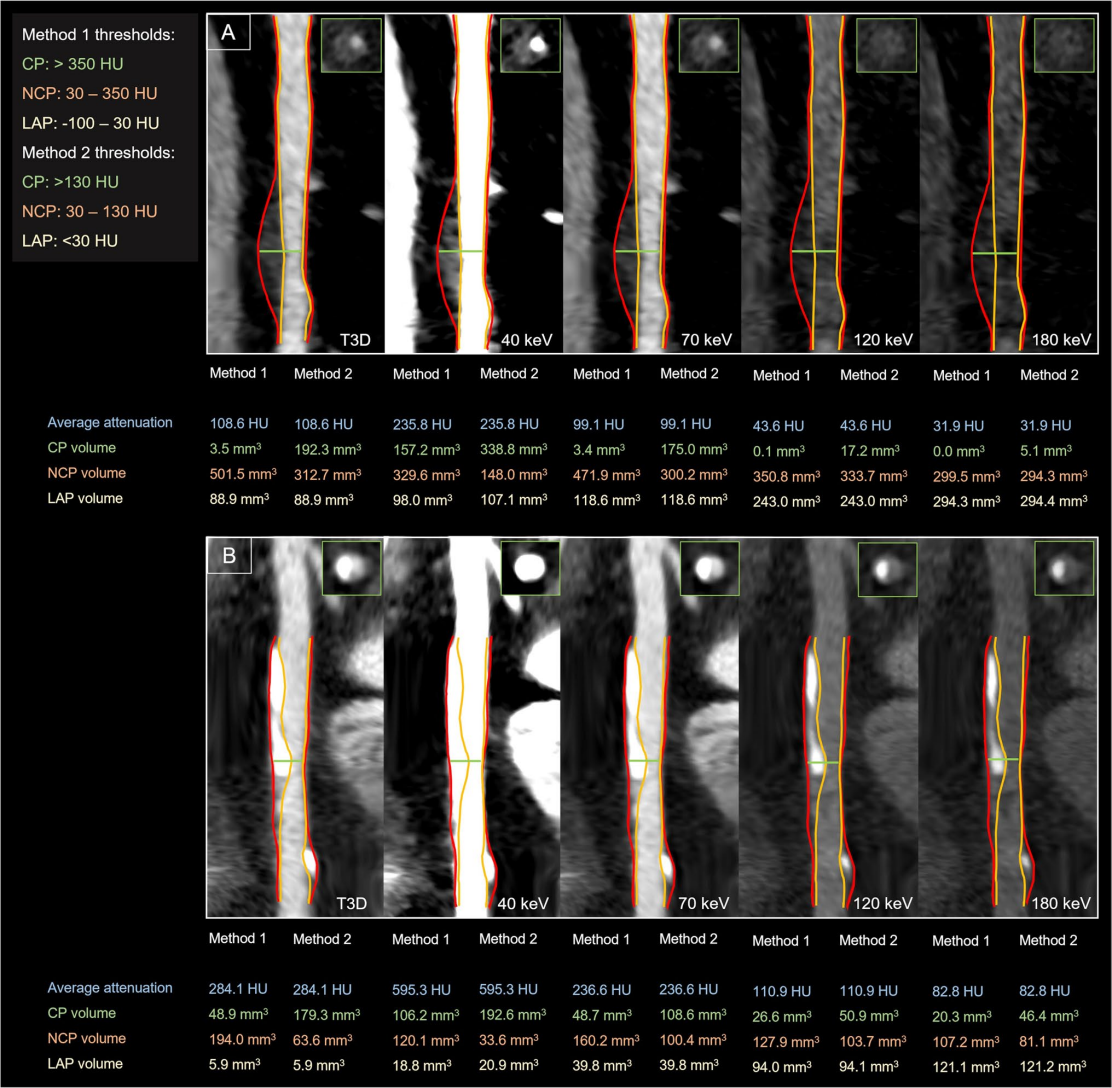
Representative CTA images of coronary plaques reconstructed in T3D and different VMI energy levels (40, 70, 120, and 180 keV). Quantitative plaque analyses of a partially calcified-predominantly non-calcified (panel **A**) and partially calcified-predominantly calcified (panel **B**) plaque are shown in T3D and different VMI reconstructions at 40, 70, 120, and 180 keV levels. The red line illustrates the border of the vessel wall and the orange line illustrates the lumen border segmented on T3D images. Corresponding cross-sectional images are also depicted at the point of the maximal narrowing of the lesion. The same window setting was applied for all represented images: window: 800; level: 250. Abbreviations: CP, calcified plaque; HU, Hounsfield unit; LAP, low-attenuation non-calcified plaque; NCP, non-calcified plaque

For all patients, VMIs were reconstructed at different energy levels from 40 to 180 keV in 10-keV increments (Fig. 1). In addition, polychromatic images at 120 kVp (T3D) were also created as reference standard for comparison. All images were reconstructed with the same settings: 0.4-mm slice thickness with 0.4-mm increment, quantitative iterative reconstruction level of 2, using a medium smooth kernel (Bv40) and a matrix of 512 × 512.

### Image quality assessment

Quantitative image quality analysis was performed for all VMI and T3D images. RadiAnt (Medixant) DICOM Viewer software (v2022.1.1) was used to measure quantitative image quality parameters by a single reader. Image noise was defined as the standard deviation (SD) of attenuation values measured by placing a circular region of interest (ROI; 200 mm^2^) in the aortic root at the level of the left main coronary ostium (SD_lumen_). Circular regions of interest (ROIs) were also placed in the coronary lumen and pericoronary fat adjacent to the analyzed lesion to measure mean attenuation in HU (HU_lumen_, HU_fat_). Artifacts and plaques were carefully avoided while manually placing ROIs. ROIs were copied from T3D images as reference and pasted to the same position on all reconstructed images for identical measurement of SD and HU values. Signal-to-noise ratio (SNR) and CNR were calculated for all reconstructed datasets, as SNR = HU_lumen_/SD_lumen_, and CNR = (HU_lumen_ − HU_fat_) / SD_lumen_.

### Quantitative plaque analysis

Coronary atherosclerotic plaque was determined on the CTA images based on prior work by Achenbach et al [9]. Quantitative plaque analysis was performed using dedicated semi-automated software (AutoPlaque 2.5; Cedars-Sinai Medical Center) by a single experienced reader (B.V.). Each coronary lesion with the highest-grade stenosis based on visual assessment was defined and analyzed per patient. We selected one lesion per patient to avoid potential intra-patient clustering effects. The centerline of the selected coronary artery was extracted; then, proximal and distal borders of the plaque were marked on the T3D images. Contouring of the vessel wall and lumen was automatic, with manual adjustment as required. Artifacts from metallic structures, beam-hardening or — in case of sequential scanning — misalignment were carefully avoided. Also, only high-quality images were used for plaque quantification that were not severely affected by motion or breathing artifacts as per exclusion criteria. Segmentation masks were copied from the T3D image to all other VMIs guaranteeing that the same voxels were analyzed on all images; therefore, potential differences in contouring on the different VMIs of the same patient did not affect our results. Our method removes the reader’s bias and focuses on the impact of different VMI reconstructions on plaque composition.

Voxels from the corresponding images were exported into the R environment (version 4.0.2) and analyzed using the Radiomics Image Analysis software package (RIA v.1.6.0) [10]. We calculated the volume of calcified plaque (CP), non-calcified plaque (NCP), and LAP. Plaque components were defined using two different methods with the following threshold ranges: method 1: LAP: − 100 to 30 HU; NCP: 30 to 350 HU; CP: > 350 HU [11, 12] and method 2: LAP: < 30 HU; NCP: 30 to 130 HU; CP: > 130 HU [13].

### Statistical analysis

Normality was assessed using Q-Q plots. Continuous variables are presented as mean and standard deviation for normally distributed data and as medians and interquartile ranges for non-normally distributed data, whereas categorical parameters are presented as frequency with percentages in the text.

We used one-way, repeated measure analysis of variances (ANOVA) and post hoc comparison analysis to compare image quality metrics, average plaque attenuation, and plaque volumes between the different monoenergetic levels. We performed two comparisons: (1) each VMI group versus T3D images as reference to answer which VMIs have significantly different values; (2) each keV group versus the next group incrementally to evaluate whether each subsequent VMI is different from the previous one. All multiple comparisons were done using pair *t*-tests and *p* values were corrected using the Bonferroni method. We calculated the relative difference between the reference T3D and all VMI reconstructions as follows: as (VMImean − T3Dmean / T3Dmean) * 100%. All statistical analyses were performed using R software (version 4.0.2) using packages: ggstatsplot (v.0.9.3) [14] and rstatix (v0.7.0). A two-sided *p* < 0.05 was considered as statistically significant.

## Results

In total, 158 patients with suspected or known CAD underwent coronary CTA using our PCCT scanner between April 2022 and June 2022. We excluded 22 patients due to inadequate image quality for plaque quantification and 85 patients without CAD. A total of 51 plaques from 51 patients were included in the analyses. Mean age was 65.1 ± 11.9 years and 68.6% were male. Common comorbidities included hypertension (80.4%), diabetes mellitus (27.5%), and dyslipidemia (52.9%). The average total plaque volume of the analyzed lesions was 270.2 ± 208.7 mm^3^ on T3D images. Mean effective radiation dose was 5.2 ± 4.3 mSv. Baseline demographic data and CT scan parameters are summarized in Table 1.

**Table 1:** Patient characteristics and CT scan parameters

Demographic data	Patient population *n* = 51
Age, years	65.1 ± 11.9
Male gender	35 (68.6%)
BMI, kg/m^2^	28.8 ± 4.7
Hypertension	41 (80%)
Diabetes mellitus	14 (28%)
Dyslipidemia	27 (53%)
Family history of premature CAD	8 (16%)
Smoking	11 (22%)
CT scan parameters	
Agatston score	444 ± 619
Total plaque volume, mm^3^	270 ± 208
DLP, mGy cm	374 ± 309
Effective dose, mSv	5.2 ± 4.3

Continuous variables are described as mean ± SD, whereas categorical variables are represented as frequencies and percentage

*BMI*, body mass index; *CAD*, coronary artery disease; *DLP*, dose-length product

### Mean plaque attenuation and quantitative image quality parameters on VMIs

**Fig. 2:**
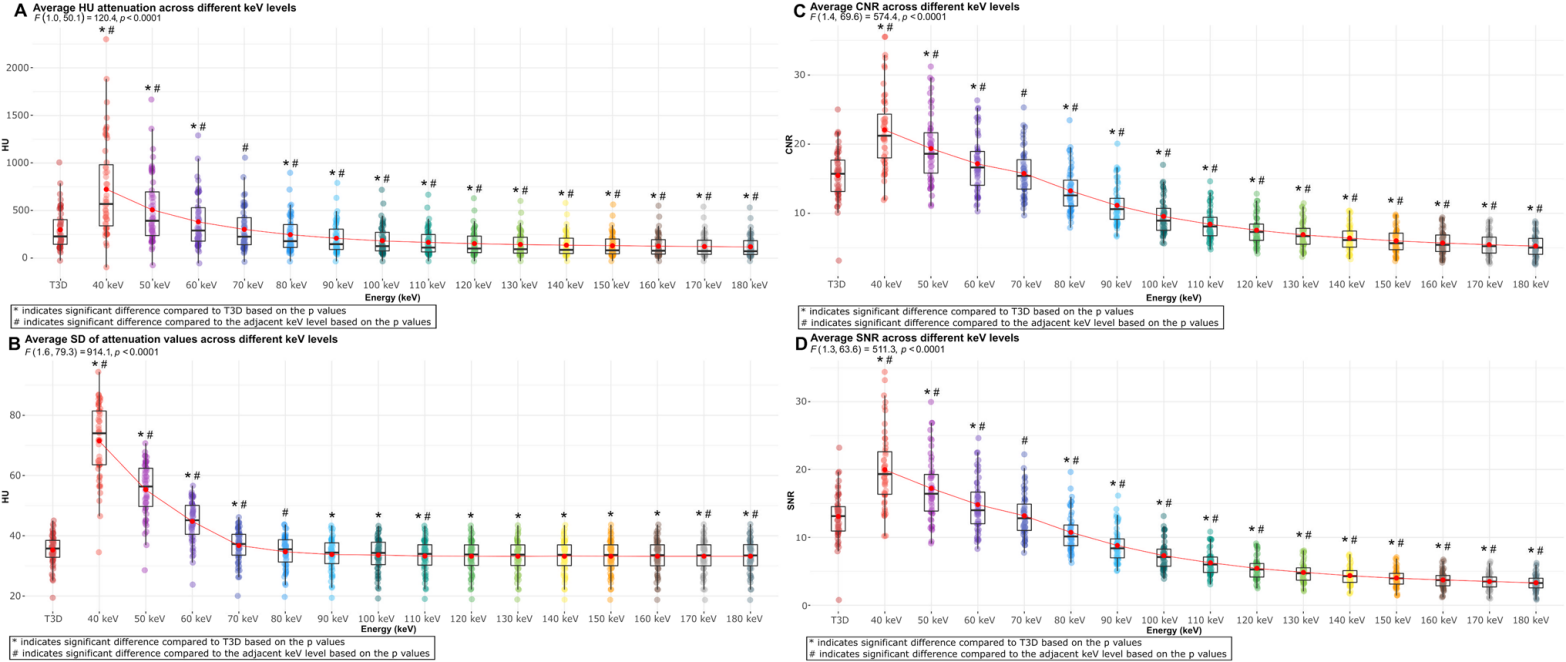
Box plots showing the distribution of plaque attenuation and quantitative image quality parameters (SD, CNR, SNR) in different VMI energy levels and T3D images. Panel **A** depicts the distribution of attenuation values across different energy levels. Panel **B** depicts the distribution of image noise based on SD across different energy levels. Panel **C** depicts the distribution CNR across different energy levels. Panel **D** depicts the distribution SNR across different energy levels. Abbreviations: CNR, contrast-to-noise ratio; HU, Hounsfield unit; SD, standard deviation; SNR, signal-to-noise ratio

Mean attenuation of the analyzed plaques was 299 ± 209 HU on T3D images. The average plaque attenuation showed a significant graded decrease with increasing keV levels (from 723 ± 501 HU on 40 keV to 120 ± 112 HU on 180 keV, *p* < 0.0001 for all). All VMIs showed a significant difference compared to T3D, except for the 70-keV images (303 ± 225 HU, *p* = 0.15) (Fig. 2A; Supplementary table 1).

There was also a decrease in image noise (SD of mean attenuation) with increasing keV levels (from 72 ± 12 HU on 40 keV to 33 ± 6 HU on 180 keV). However, a significant difference in image noise between adjacent keV levels was not uniformly observed. Images reconstructed at 80 keV showed similar image noise compared to T3D images (35 ± 5 HU vs. 35 ± 5 HU, respectively; *p* = 0.74) (Fig. 2B).

Tendencies for CNR (highest at 40 keV: 22.1 ± 5.6 and lowest at 180 keV: 5.3 ± 1.6; each value significantly different from the adjacent keV level) and SNR (highest at 40 keV: 20.0 ± 5.5 and lowest at 180 keV: 3.3 ± 1.2; each value significantly different from the adjacent keV level) were similar to those for mean attenuation. T3D images yielded similar image quality based on CNR and SNR as compared with 70-keV VMI reconstructions (15.5 ± 3.7 vs. 15.8 ± 3.5, *p* = 0.32 for CNR and 13.1 ± 3.6 vs. 13.2 ± 3.2, *p* = 0.69 for SNR, respectively) (Fig. 2C and D, respectively).

### Changes in plaque volumes using different VMI reconstructions

**Fig. 3:**
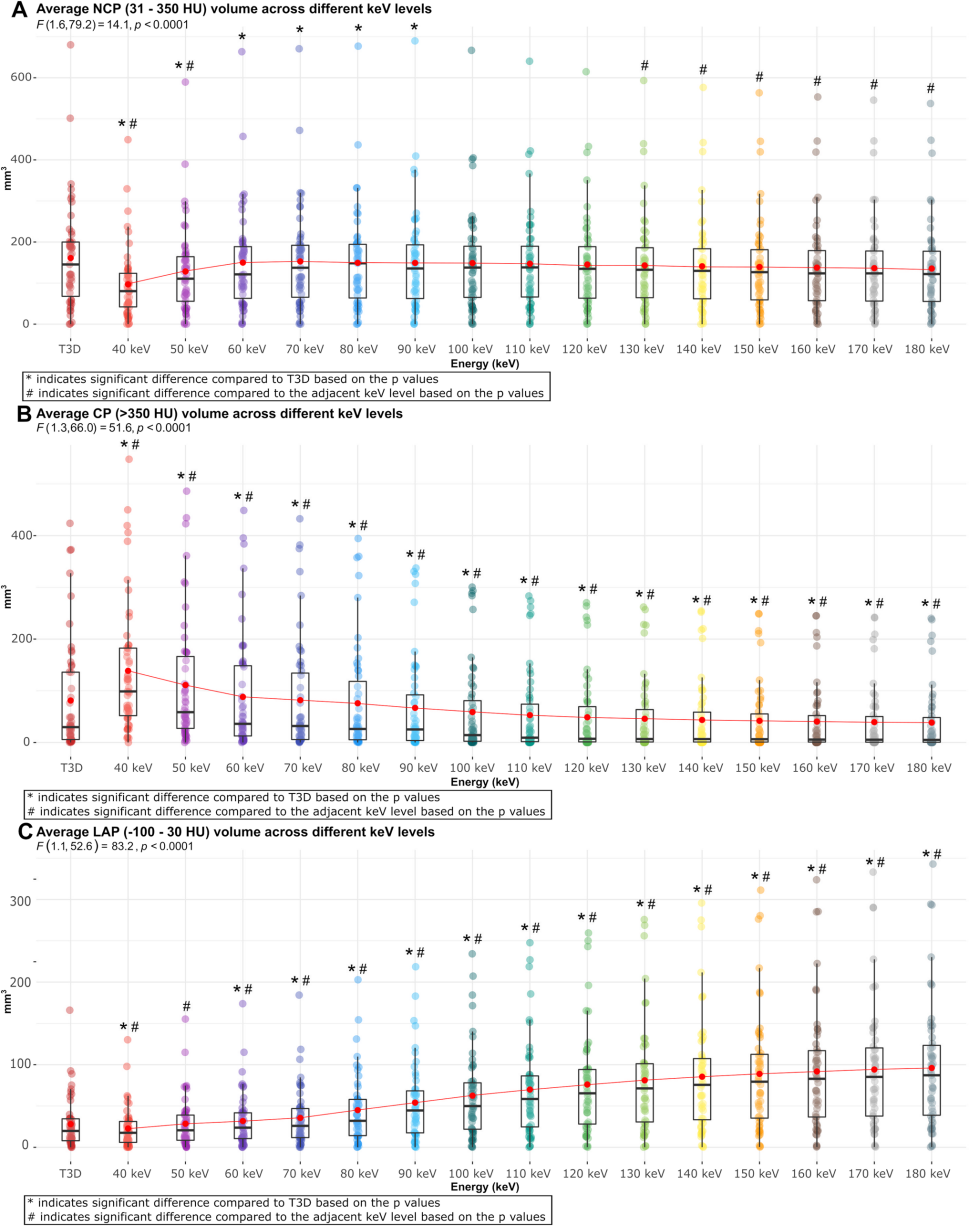
Box plots showing the distribution of plaque volumes in different VMI energy levels and T3D images using the thresholds of method 1 for plaque characterization. Panel **A** shows the distribution of NCP volume across different energy levels using threshold of − 100 to 350 HU. Panel **B** shows the distribution of CP volume across different energy levels using the threshold of > 350 HU. Panel **C** shows the distribution of LAP volume across different energy levels using the threshold of − 100 to 30 HU. Abbreviations: CP, calcified plaque; HU, Hounsfield unit; LAP, low-attenuation non-calcified plaque; NCP, non-calcified plaque; SD, standard deviation

**Fig. 4:**
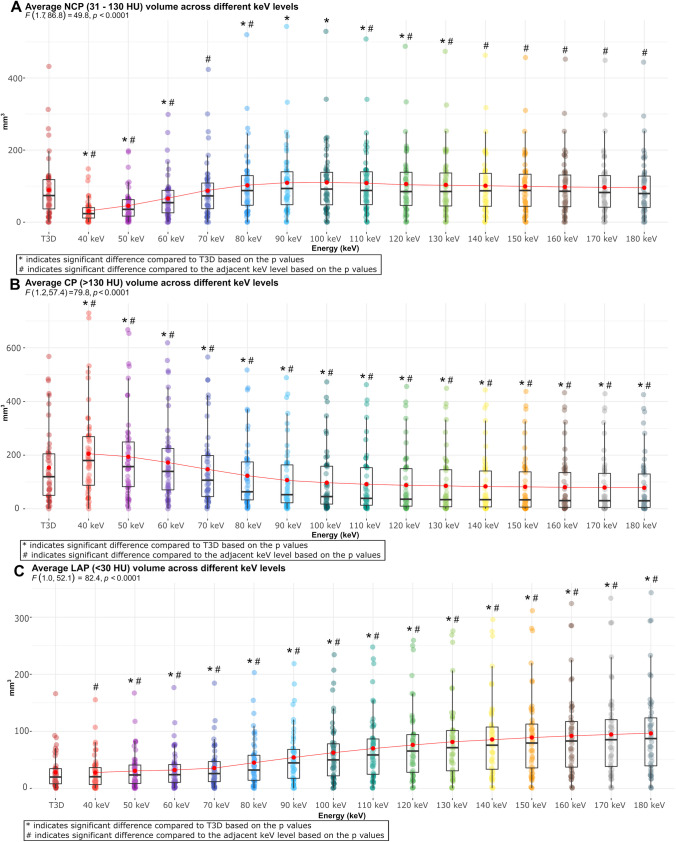
Box plots showing the distribution of plaque volumes in different VMI energy levels and T3D images using the thresholds of method 2 for plaque characterization. Panel **A** shows the distribution of NCP volume across different energy levels using threshold of < 130 HU. Panel **B** shows the distribution of CP volume across different energy levels using the threshold of > 130 HU. Panel **C** shows the distribution of LAP volume across different energy levels using the threshold of < 30 HU. Abbreviations: CP, calcified plaque; HU, Hounsfield unit; LAP, low-attenuation non-calcified plaque; NCP, non-calcified plaque; SD, standard deviation

We applied two threshold settings for plaque quantification: method 1: LAP: − 100 to 30 HU; NCP: 30 to 350 HU; CP: > 350 HU [11, 12] and method 2: LAP: < 30 HU; NCP: 30 to 130 HU; CP: > 130 HU [13] (Figs. 3 and 4, respectively).

Using method 1, mean NCP volume was 161.0 ± 126.3 mm^3^ on T3D images. The average NCP volume showed an increase up to 70 keV, followed by a decrease with each subsequent increment in VMI energy level. A significant difference in NCP volume between keV levels was not observed at every step. The lowest value was found using 40 keV (96.9 ± 86.8 mm^3^), whereas the highest was seen using 70 keV (152.8 ± 122.0 mm^3^). Mean NCP volume measured on 100–180-keV reconstructions did not differ significantly from T3D images (*p* > 0.05 for all) (Fig. 3A). The average CP volume showed a significant graded decrease with increasing keV levels, from 138.7 ± 126.4 mm^3^ on 40 keV to 38.5 ± 64.6 mm^3^ on 180 keV (*p* < 0.001 for all). Mean CP volume measured on each VMI reconstruction differed significantly from the reference T3D images (*p* < 0.05 for all) (Fig. 3B). An increasing LAP volume for each increment in keV level was observed, with a significant difference between each step (from 22.8 ± 24.9 mm^3^ on 40 keV to 96.0 ± 76.3 mm^3^ on 180 keV; *p* < 0.0001 for all). Mean LAP volume differed significantly between T3D and VMI reconstructions, except for 50-keV images (28.0 ± 30.8 mm^3^ and 28.6 ± 30.1 mm^3^, respectively, *p* = 0.63) (Fig. 3C; Supplementary table 2).

Method 2 yielded similar findings for the assessment of plaque volumes. Mean NCP volume showed an initial increase up to 100 keV, followed by a decrease with increasing keV levels, with no significant difference between each adjacent keV level. Average NCP volume on T3D was comparable with 70- and 140–180-keV energy levels (*p* > 0.05 for all) (Fig. 4A). Similarly, mean CP volume showed a decreasing tendency with significant difference between each adjacent keV level (*p* < 0.01 for all). Mean CP volume measured on each VMI reconstructions differed significantly from the reference T3D images (*p* < 0.001 for all) (Fig. 4B). Also, an increasing tendency of LAP volume was observed by increasing keV levels, with significant difference between each adjacent VMI (*p* < 0.05 for all). When measuring LAP volume, all VMIs showed a significant difference compared to T3D, except for 40 keV (*p* = 0.65) (Fig. 4C; Supplementary table 3).

### Relative difference between T3D and VMI reconstructions

The relative difference regarding attenuation and image quality parameters between the reference standard T3D and VMI reconstructions are summarized in Table 2. Regarding plaque volumes using thresholds of method 1 for plaque characterization, the highest difference for CP and NCP volume was measured on 40-keV images compared to T3D (70.8% and − 39.8%, respectively, *p* < 0.0001), whereas the lowest relative difference was measured using 70-keV images (0.9% and − 5.1%, respectively, *p* < 0.0001). Mean LAP volume showed the largest discrepancy on 180-keV reconstruction with 242.5% relative difference (*p* < 0.0001) and the smallest on 50-keV images (1.9%, *p* = 0.63) (Table 3).

**Table 2:** Difference in CT attenuation and quantitative image quality parameters (SD, CNR, SNR) for different energy levels compared to T3D

Energy level (keV)	Attenuation (HU)		Image noise (SD)		CNR		SNR	
	Difference to T3D (%)	95% CI	Difference to T3D (%)	95% CI	Difference to T3D (%)	95% CI	Difference to T3D (%)	95% CI
40	142.0	114.3 to 169.6	102.7	95.8 to 109.7	42.6	36.1 to 49.0	52.7	45.1 to 60.3
50	70.1	55.8 to 84.5	56.8	52.4 to 61.1	25.2	19.8 to 30.5	31.7	25.5 to 37.9
60	27.9	21.5 to 34.4	27.0	24.2 to 29.7	11.0	6.5 to 15.4	13.4	8.4 to 18.4
70	1.3*	− 0.5 to 3.2	4.2	2.3 to 6.1	1.8*	− 1.8 to 5.4	0.8*	− 3.2 to 4.8
80	− 17.4	− 19.7 to − 15.1	− 1.7*	− 4.1 to 0.6	− 14.4	− 18.0 to − 10.8	− 17.9	− 21.8 to − 13.9
90	− 29.9	− 34.2 to − 25.6	− 4.3	− 6.9 to − 1.7	− 28.0	− 31.8 to − 24.1	− 32.6	− 36.8 to − 28.4
100	− 38.3	− 44.1 to − 32.5	− 4.9	− 7.6 to − 2.2	− 38.3	− 42.3 to − 34.3	− 44.1	− 48.7 to − 39.5
110	− 44.3	− 51.1 to − 37.5	− 5.7	− 8.6 to − 2.9	− 45.6	− 49.8 to − 41.3	− 52.2	− 57.2 to − 47.2
120	− 48.6	− 56.2 to − 41.0	− 5.9	− 8.8 to − 3.0	− 51.2	− 55.7 to − 46.6	− 58.4	− 63.7 to − 53.0
130	− 51.8	− 60.0 to − 43.7	− 5.9	− 8.9 to − 3.0	− 55.4	− 60.1 to − 50.7	− 63.0	− 68.7 to − 57.4
140	− 54.3	− 62.9 to − 45.7	− 6.0	− 8.9 to − 3.0	− 58.6	− 63.5 to − 53.7	− 66.6	− 72.5 to − 60.7
150	− 56.2	− 65.1 to − 47.2	− 5.9	− 8.9 to − 3.0	− 61.1	− 66.1 to − 56.1	− 69.4	− 75.4 to − 63.3
160	− 57.6	− 66.9 to − 48.4	− 5.9	− 8.8 to − 2.9	− 63.1	− 68.2 to − 58.0	− 71.6	− 77.8 to − 65.4
170	− 58.8	− 68.3 to − 49.4	− 5.9	− 8.8 to − 2.9	− 64.7	− 69.9 to − 59.5	− 73.3	− 79.6 to − 67.0
180	− 59.8	− 69.4 to − 50.2	− 5.8	− 8.8 to − 2.9	− 66.0	− 71.2 to − 60.7	− 74.7	− 81.1 to − 68.3

Differences and 95% confidence intervals (CI) are from pairwise *t*-tests

^*^ indicates no statistical significance compared to T3D based on *p* value

*CNR*, contrast-to-noise ratio; *HU*, Hounsfield unit; *SD*, standard deviation; *SNR*, signal-to-noise ratio

**Table 3:** Difference in CP, NCP, and LAP volumes based on the thresholds of method 1 (CP > 350 HU; NCP: 31 to 350 HU; LAP: − 100 to 30 HU) for different energy levels compared to T3D

Energy level (keV)	CP (> 350 HU)		NCP (31 to 350 HU)		LAP (− 100 to 30 HU)	
	Difference to T3D (%)	95% CI	Difference to T3D (%)	95% CI	Difference to T3D (%)	95% CI
40	70.8	54.2 to 87.5	− 39.8	− 48.1 to − 31.5	− 18.8	− 29.0 to − 8.6
50	36.9	28.2 to 45.6	− 20.3	− 25.2 to − 15.5	1.9*	− 5.8 to 9.6
60	8.7	5.4 to 12.0	− 6.9	− 8.9 to − 4.9	13.6	7.5 to 19.6
70	0.9	0.1 to 1.6	− 5.1	− 6.4 to − 3.8	26.8	19.8 to 33.7
80	− 6.5	− 9.4 to − 3.6	− 7.3	− 9.7 to − 4.9	60.5	46.9 to 74.0
90	− 17.5	− 24.9 to − 10.0	− 7.4	− 11.4 to − 3.4	92.9	72.5 to 113.3
100	− 27.5	− 38.2 to − 16.8	− 7.6*	− 13.2 to − 2.0	122.9	96.1 to 149.7
110	− 34.8	− 47.6 to − 21.9	− 8.6*	− 15.3 to − 1.8	149.3	116.9 to 181.8
120	− 39.8	− 54.2 to − 25.5	− 9.8*	− 17.5 to − 2.2	171.2	133.9 to 208.4
130	− 43.4	− 58.9 to − 28.0	− 11.2*	− 19.6 to − 2.8	189.3	148.0 to 230.5
140	− 46.3	− 62.5 to − 30.0	− 12.4*	− 21.4 to − 3.5	204.7	160.2 to 249.2
150	− 48.3	− 65.3 to − 31.4	− 13.6*	− 23.0 to − 4.1	217.0	170.0 to 263.9
160	− 50.1	− 67.6 to − 32.6	− 14.5*	− 24.3 to − 4.7	227.4	178.4 to 276.4
170	− 51.4	− 69.4 to − 33.5	− 15.3*	− 25.4 to − 5.2	235.7	185.1 to 286.3
180	− 52.5	− 70.9 to − 34.2	− 15.9*	− 26.3 to − 5.5	242.5	190.5 to 294.6

Differences and 95% confidence intervals (CI) are from pairwise *t*-tests

^*^ indicates no statistical significance compared to T3D based on *p* value

*CP*, calcified plaque; *HU*, Hounsfield unit; *LAP*, low-attenuation non-calcified plaque; *NCP*, non-calcified plaque

Applying method 2 for plaque quantification, the greatest difference in CP volume was observed between 180 keV and T3D, with a relative difference of − 48.9% (*p* < 0.0001), while the lowest relative difference of CP volume was observed at 70-keV images (− 4.0%, *p* < 0.001). For NCP volume as compared with T3D, the greatest and smallest differences were observed using 40 (− 65.6%, *p* < 0.0001) and 70 keV (− 1.5%, *p* = 0.54), respectively. The largest relative difference for LAP volume was seen on 180-keV images (243.3%, *p* < 0.0001), and the lowest on 40-keV images (− 1.9%, *p* = 0.65) (Table 4).

**Table 4:** Difference in CP, NCP, and LAP volumes based on the thresholds of method 2 (CP > 130 HU; NCP: 31 to 130 HU; LAP: < 30 HU) for different energy levels compared to T3D

Energy level (keV)	CP (> 130 HU)		NCP (31 to 130 HU)		LAP (< 30 HU)	
	Difference to T3D (%)	95% CI	Difference to T3D (%)	95% CI	Difference to T3D (%)	95% CI
40	33.6	23.3 to 44.0	− 65.6	− 82.4 to − 48.8	− 1.9*	− 10.1 to 6.4
50	26.5	19.3 to 33.6	− 48.9	− 61.6 to − 36.2	9.6	2.2 to 17.0
60	12.5	8.4 to 16.6	− 26.2	− 33.4 to − 18.9	14.4	8.4 to 20.4
70	− 4.0	− 5.9 to − 2.1	− 1.5*	− 3.9 to 0.9	26.8	19.8 to 33.8
80	− 19.7	− 24.1 to − 15.4	15.0	9.8 to 20.1	60.5	47.0 to 74.1
90	− 30.4	− 36.7 to − 24.0	23.1	16.1 to 30.1	93.0	72.6 to 113.5
100	− 36.5	− 44.1 to − 29.0	24.2	16.0 to 32.5	123.1	96.2 to 149.9
110	− 40.2	− 48.6 to − 31.8	22.2	13.1 to 31.3	149.6	117.1 to 182.1
120	− 42.6	− 51.5 to − 33.7	19.4	9.7 to 29.1	171.5	134.2 to 208.9
130	− 44.3	− 53.5 to − 35.0	16.5	6.3 to 26.7	189.7	148.4 to 231.0
140	− 45.6	− 55.1 to − 36.0	13.9*	3.4 to 24.4	205.2	160.7 to 249.8
150	− 46.7	− 56.5 to − 36.8	11.8*	1.1 to 22.6	217.6	170.6 to 264.6
160	− 47.6	− 57.6 to − 37.5	10.1*	− 0.9 to 21.1	228.1	179.0 to 277.2
170	− 48.3	− 58.5 to − 38.1	8.7*	− 2.5 to 19.9	236.5	185.7 to 287.2
180	− 48.9	− 59.3 to − 38.5	7.7*	− 3.7 to 19.1	243.3	191.2 to 295.5

Differences and 95% confidence intervals (CI) are from pairwise *t*-tests

^*^ indicates no statistical significance compared to T3D based on *p* value

*CP*, calcified plaque; *HU*, Hounsfield unit; *LAP*, low-attenuation non-calcified plaque; *NCP*, non-calcified plaque

## Discussion

In our study, we demonstrate that utilizing different VMI energy levels from PCCT for the analysis of coronary artery plaques leads to substantial changes in attenuation values and corresponding plaque component volumes. Our primary findings are as follows: (1) Low-energy images (40–70 keV) improved CNR and resulted in higher CP but lower NCP and LAP volumes, however also increased image noise; (2) CP volume quantified on 70 keV exhibited the lowest relative difference compared to T3D images; (3) No significant differences were observed in NCP volume using higher VMI levels (100–180 keV); (4) LAP volume was not significantly different on low-energy images (40–50 keV) when compared to T3D as reference.

Coronary CTA is a well-established non-invasive modality for the assessment of CAD in patients presenting with stable chest pain [15]. In addition to luminal stenosis, CTA uniquely allows for the evaluation of overall plaque burden and high-risk plaque characteristics, which are strong predictors of subsequent cardiovascular events [1]. Moreover, quantitative plaque analysis provides comprehensive assessment of coronary plaque volume and composition. A growing body of evidence suggests the additional prognostic value of quantitative plaque metrics over visual assessment alone [16]. In particular, LAP (defined using a fixed HU threshold of 30) burden independently predicted myocardial infarction at 5 years’ follow-up in patients with stable chest pain [2]. Accordingly, using CTA for the quantitative evaluation of CAD can improve risk stratification by identifying patients at high risk [17].

A novel dual-source PCCT system has recently been introduced in clinical practice with the potential to overcome several limitations of conventional CT scanners. Photon-counting detectors directly generate electronical signal proportional to photon energy by measuring the energy of each individual photon reaching the detector. Compared to traditional energy-integrating detectors, PCCT is characterized by superior spatial resolution, reduction of image noise, and beam-hardening artifacts [4]. In addition, this technology enables advanced tissue characterization with the use of virtual monoenergetic reconstructions and therefore may provide improved plaque assessment. However, VMIs also change the CT attenuation values which may affect plaque characterization.

In general, the benefit of lower keV images is the increased contrast between the coronary lumen and vessel wall, aiding better discrimination of coronary plaques and intraluminal contrast. Despite showing higher image noise, lower keV level images yield better image quality based on CNR and SNR as demonstrated also in previous phantom studies [18]. On the other hand, higher keV level images decrease blooming and image noise, which can enhance calcified plaque analysis. However, higher keV levels result in decreased CNR and SNR. To utilize the advantages from both high- and low-energy level images, it would be advantageous to view the different keV images side-by-side and have the ability to manually edit segmentation contours on either image to minimize the effects of different artefacts. Ohta et al demonstrated using dual-energy CT datasets that different VMI energy levels showed the highest CNR and SNR for each coronary plaque component. In line with our results, these findings also suggested that different VMIs should be used simultaneously for coronary plaque assessment to apply the advantage of each energy levels [19].

As different keV images change the attenuation values of the voxels, it is also important to consider how these changes affect the quantification of plaque composition. Based on our results, plaque compositional volumes defined using fixed threshold settings on T3D images yielded similar results in different VMI reconstructions for the different plaque components. This is due to the fact that attenuation values change on different VMIs as a function of the tissue composition of the given voxel [20–22]. Therefore, fixed threshold setting will not work on VMIs which is emphasized by our results as we observed significantly different plaque volumes on almost all VMIs as compared to T3D as a reference. Furthermore, one universal correction factor is not enough as we show that the relative difference in plaque volumes is different for each component on the same VMI. Therefore, to utilize the information from VMIs and have comparable results between future studies and previous investigations, we need to develop standardized protocols and adaptive correction factors which may allow conversions between volumetric estimates done using different VMIs and/or conventional images.

To the best of our knowledge, no previous study has investigated the influence of different VMI energy levels on coronary plaque composition using PCCT datasets. Symons et al examined the impact of different VMI energy levels on coronary plaque segmentation and quantification using 3rd-generation dual-source CT scanner. They reported similar tendencies in image quality parameters to our results, with better CNR and SNR, and higher image noise on lower keV images. Although a different method was applied during plaque analysis — as segmentation was performed individually on every reconstruction — similar tendencies were also observed regarding plaque types [23].

We acknowledge the limitations of our study. First, this was a single-center study focusing on plaque quantification in a relatively small population of stable chest pain patients. However, we believe that the sample was large enough to evaluate the trends in using different VMI reconstructions for plaque assessment. Second, we only used a single software for plaque segmentation. Furthermore, there are additional parameters that could alter plaque volumes (such as slice thickness, iterative reconstruction, kernels) — that has previously been investigated — which were not part of our analysis [24, 25]. Also, we had no reference standard. Histology or intravascular imaging was not available in these individuals. Nevertheless, T3D polychromatic images are considered to be comparable to conventional 120-kV polychromatic images on energy-integrating detector CT scanner and the methods used have been validated to intravascular imaging and tested in large cohorts on conventional images [2, 26, 27]. Using different flow rates, contrast media concentrations and dosing could lead to different attenuation values in the coronaries. However, our protocol only allows for small changes in the given contrast media dose using the same concentration. Furthermore, we evaluated changes within the same patient on different VMIs, and thus, the potential effect of contrast administration on plaque volumes should be consistent within each patient [28].

In conclusion, low-energy monoenergetic reconstructions significantly alter plaque attenuation and plaque volumes with fixed plaque attenuation thresholds. Therefore, caution is required when using different VMI reconstructions and fixed plaque attenuation thresholds for plaque characterization. New standards and protocols are required to determine which monoenergetic or polyenergetic images are optimal to derive plaque volumes. This will aid the adoption of PCCT plaque analysis in both the clinical and research setting.

### Supplementary Information

Below is the link to the electronic supplementary material.

Supplementary file1 (PDF 73 KB)

## Abbreviations

CAD: Coronary artery disease
CNR: Contrast-to-noise ratio
CP: Calcified plaque
CTA: Computed tomography angiography
HU: Hounsfield unit
LAP: Low-attenuation non-calcified plaque
NCP: Non-calcified plaque
PCCT: Photon-counting computed tomography
ROI: Region of interest
SD: Standard deviation
SNR: Signal-to-noise ratio
VMI: Virtual monoenergetic image

## Declarations

### Guarantor

The scientific guarantor of this publication is Márton Kolossváry MD PhD.

### Statistics and biometry

One of the authors has significant statistical expertise.

### Methodology

• prospective

• cross-sectional study

• performed at one institution

## References

[1] Motoyama S, Ito H, Sarai M (2015). Plaque Characterization by Coronary Computed Tomography Angiography and the Likelihood of Acute Coronary Events in Mid-Term Follow-Up. J Am Coll Cardiol.

[2] Williams MC, Kwiecinski J, Doris M (2020). Low-Attenuation Noncalcified Plaque on Coronary Computed Tomography Angiography Predicts Myocardial Infarction: Results From the Multicenter SCOT-HEART Trial (Scottish Computed Tomography of the HEART). Circulation.

[3] Sandfort V, Persson M, Pourmorteza A, Noel PB, Fleischmann D, Willemink MJ (2021). Spectral photon-counting CT in cardiovascular imaging. J Cardiovasc Comput Tomogr.

[4] Willemink MJ, Persson M, Pourmorteza A, Pelc NJ, Fleischmann D (2018). Photon-counting CT: Technical Principles and Clinical Prospects. Radiology.

[5] Boussel L, Coulon P, Thran A (2014). Photon counting spectral CT component analysis of coronary artery atherosclerotic plaque samples. Br J Radiol.

[6] Grant KL, Flohr TG, Krauss B, Sedlmair M, Thomas C, Schmidt B (2014). Assessment of an advanced image-based technique to calculate virtual monoenergetic computed tomographic images from a dual-energy examination to improve contrast-to-noise ratio in examinations using iodinated contrast media. Invest Radiol.

[7] Abbara S, Blanke P, Maroules CD (2016). SCCT guidelines for the performance and acquisition of coronary computed tomographic angiography: A report of the society of Cardiovascular Computed Tomography Guidelines Committee: Endorsed by the North American Society for Cardiovascular Imaging (NASCI). J Cardiovasc Comput Tomogr.

[8] Karady J, Panajotu A, Kolossvary M (2017). The effect of four-phasic versus three-phasic contrast media injection protocols on extravasation rate in coronary CT angiography: a randomized controlled trial. Eur Radiol.

[9] Achenbach S, Moselewski F, Ropers D (2004). Detection of calcified and noncalcified coronary atherosclerotic plaque by contrast-enhanced, submillimeter multidetector spiral computed tomography: a segment-based comparison with intravascular ultrasound. Circulation.

[10] Kolossvary M, Karady J, Szilveszter B (2017). Radiomic Features Are Superior to Conventional Quantitative Computed Tomographic Metrics to Identify Coronary Plaques With Napkin-Ring Sign. Circ Cardiovasc Imaging.

[11] Kolossvary M, Bluemke DA, Fishman EK (2022). Temporal assessment of lesion morphology on radiological images beyond lesion volumes-a proof-of-principle study. Eur Radiol.

[12] Kolossvary M, Gerstenblith G, Bluemke DA (2021). Contribution of Risk Factors to the Development of Coronary Atherosclerosis as Confirmed via Coronary CT Angiography: A Longitudinal Radiomics-based Study. Radiology.

[13] Ferencik M, Mayrhofer T, Puchner SB (2015). Computed tomography-based high-risk coronary plaque score to predict acute coronary syndrome among patients with acute chest pain–Results from the ROMICAT II trial. J Cardiovasc Comput Tomogr.

[14] Patil I (2021). Visualizations with statistical details: The ‘ggstatsplot’ approach. J Open Source Softw.

[15] Writing Committee M, Gulati M, Levy PD (2021). 2021 AHA/ACC/ASE/CHEST/SAEM/SCCT/SCMR Guideline for the Evaluation and Diagnosis of Chest Pain: A Report of the American College of Cardiology/American Heart Association Joint Committee on Clinical Practice Guidelines. J Am Coll Cardiol.

[16] Nadjiri J, Hausleiter J, Jahnichen C (2016). Incremental prognostic value of quantitative plaque assessment in coronary CT angiography during 5 years of follow up. J Cardiovasc Comput Tomogr.

[17] Chang HJ, Lin FY, Lee SE (2018). Coronary Atherosclerotic Precursors of Acute Coronary Syndromes. J Am Coll Cardiol.

[18] Sartoretti T, McDermott M, Mergen V (2023). Photon-counting detector coronary CT angiography: impact of virtual monoenergetic imaging and iterative reconstruction on image quality. Br J Radiol.

[19] Ohta Y, Kitao S, Watanabe T, Kishimoto J, Yamamoto K, Ogawa T (2017). Evaluation of image quality of coronary artery plaque with rapid kVp-switching dual-energy CT. Clin Imaging.

[20] Rajendran K, Petersilka M, Henning A (2022). First Clinical Photon-counting Detector CT System: Technical Evaluation. Radiology.

[21] Mergen V, Ried E, Allmendinger T (2022). Epicardial Adipose Tissue Attenuation and Fat Attenuation Index: Phantom Study and In Vivo Measurements With Photon-Counting Detector CT. AJR Am J Roentgenol.

[22] Eberhard M, Mergen V, Higashigaito K (2021). Coronary Calcium Scoring with First Generation Dual-Source Photon-Counting CT-First Evidence from Phantom and In-Vivo Scans. Diagnostics (Basel).

[23] Symons R, Choi Y, Cork TE (2018). Optimized energy of spectral coronary CT angiography for coronary plaque detection and quantification. J Cardiovasc Comput Tomogr.

[24] Mergen V, Eberhard M, Manka R, Euler A, Alkadhi H (2022). First in-human quantitative plaque characterization with ultra-high resolution coronary photon-counting CT angiography. Front Cardiovasc Med.

[25] Mergen V, Sartoretti T, Baer-Beck M (2022). Ultra-High-Resolution Coronary CT Angiography With Photon-Counting Detector CT: Feasibility and Image Characterization. Invest Radiol.

[26] Matsumoto H, Watanabe S, Kyo E (2019). Standardized volumetric plaque quantification and characterization from coronary CT angiography: a head-to-head comparison with invasive intravascular ultrasound. Eur Radiol.

[27] Meah MN, Singh T, Williams MC (2021). Reproducibility of quantitative plaque measurement in advanced coronary artery disease. J Cardiovasc Comput Tomogr.

[28] Cademartiri F, Mollet NR, Runza G (2005). Influence of intracoronary attenuation on coronary plaque measurements using multislice computed tomography: observations in an ex vivo model of coronary computed tomography angiography. Eur Radiol.

