# Supplementary material for: Impact of virtual monoenergetic levels on coronary plaque volume components using photon-counting computed tomography
Source: Eur Radiol. 2023 Jul 24;33(12):8528–39. doi: 10.1007/s00330-023-09876-7 (PMC10667372; doi:10.1007/s00330-023-09876-7)
Supplement: Supplementary file 1 — Supplementary file1 (PDF 73 KB) [file 330_2023_9876_MOESM1_ESM.pdf]

Impact of Virtual Monoenergetic Levels on Coronary Plaque Volume Components using Photon-Counting Computed Tomography

ELECTRONIC SUPPLEMENTARY MATERIAL

Supplementary table 1. Average attenuation and image quality values (SD, CNR, SNR) across different VMI energy levels and T3D images.

Energy level (keV)	Attenuation (HU)		Image noise (SD)		CNR		SNR	
	Mean	SD	Mean	SD	Mean	SD	Mean	SD
T3D	299	209	35	5	15.5	3.7	13.1	3.6
40	723	501	72	12	22.1	5.6	20.0	5.5
50	509	361	55	9	19.4	4.9	17.2	4.7
60	382	277	45	7	17.2	4.0	14.8	3.8
70	303	225	37	6	15.8	3.5	13.2	3.2
80	247	190	35	5	13.2	3.2	10.7	2.8
90	210	167	34	5	11.1	2.9	8.8	2.4
100	184	151	34	6	9.5	2.5	7.3	2.1
110	167	140	33	6	8.4	2.3	6.3	1.8
120	154	132	33	6	7.6	2.1	5.4	1.6
130	144	127	33	6	6.9	1.9	4.8	1.5
140	137	122	33	6	6.4	1.8	4.4	1.4
150	131	119	33	6	6.0	1.8	4.0	1.3
160	127	116	33	6	5.7	1.7	3.7	1.3
170	123	114	33	6	5.5	1.7	3.5	1.2
180	120	112	33	6	5.3	1.6	3.3	1.2

CNR: Contrast-to-noise ratio; CP: Calcified plaque; HU: Hounsfield unit; SD: Standard deviation; SNR: Signal-to-noise ratio

Supplementary table 2. Average plaque volumes and their proportion to total plaque volume (%) across different VMI energy levels and T3D images using the thresholds of method 1 for plaque characterization.

CP: Calcified plaque; HU: Hounsfield unit; LAP: Low-attenuation non-calcified plaque; NCP: Non-calcified plaque; SD: Standard deviation; TPV: Total plaque volume on T3D images

Energy level (keV)	CP volume (>350 HU)			NCP volume (31 - 350 HU)			LAP volume (-100 - 30 HU)		
	Mean (mm^3)	SD (mm^3)	Proportion of mean to TPV (%)	Mean (mm^3)	SD (mm^3)	Proportion of mean to TPV (%)	Mean (mm^3)	SD (mm^3)	Proportion of mean to TPV (%)
T3D	81.2	110.1	30.0	161.0	126.3	59.6	28.0	30.8	10.4
40	138.7	126.4	51.3	96.9	86.8	35.9	22.8	24.9	8.4
50	111.1	121.5	41.1	128.3	106.9	47.5	28.6	30.1	10.6
60	88.2	113.3	32.6	149.9	120.1	55.5	31.8	32.9	11.8
70	81.9	111.4	30.3	152.8	122.0	56.6	35.5	35.0	13.1
80	75.9	105.1	28.1	149.3	120.7	55.3	45.0	41.4	16.7
90	67.0	95.6	24.8	149.1	123.0	55.2	54.1	47.6	20.0
100	58.9	87.1	21.8	148.8	122.6	55.1	62.5	53.3	23.1
110	53.0	81.0	19.6	147.2	120.9	54.5	69.9	58.4	25.9
120	48.8	76.5	18.1	145.2	118.9	53.7	76.0	62.7	28.1
130	45.9	73.1	17.0	143.0	117.1	52.9	81.1	66.3	30.0
140	43.6	70.4	16.1	141.0	115.5	52.2	85.4	69.3	31.6
150	41.9	68.5	15.5	139.2	114.3	51.5	88.9	71.5	32.9
160	40.5	66.9	15.0	137.7	113.3	51.0	91.8	73.5	34.0
170	39.4	65.6	14.6	136.4	112.6	50.5	94.1	75.0	34.8
180	38.5	64.6	14.3	135.4	111.9	50.1	96.0	76.3	35.5

Supplementary table 3. Average plaque volumes and their proportion to total plaque volume (%) across different VMI energy levels and T3D images using the thresholds of method 2 for plaque characterization.

Energy level (keV)	CP volume (>130 HU)			NCP volume (31 - 130 HU)			LAP volume (<30 HU)		
	Mean (mm^3)	SD (mm^3)	Proportion of mean to TPV (%)	Mean (mm^3)	SD (mm^3)	Proportion of mean to TPV (%)	Mean (mm^3)	SD (mm^3)	Proportion of mean to TPV (%)
T3D	153.4	138.4	56.8	88.8	82.2	32.9	28.1	30.8	10.4
40	205.0	164.2	75.9	30.6	31.3	11.3	27.5	29.9	10.2
50	194.0	161.6	71.8	45.4	43.9	16.8	30.7	32.3	11.4
60	172.6	150.3	63.9	65.6	60.5	24.3	32.1	33.3	11.9
70	147.2	138.3	54.5	87.4	79.4	32.4	35.6	35.1	13.2
80	123.1	127.5	45.6	102.1	90.3	37.8	45.0	41.5	16.7
90	106.8	120.9	39.5	109.3	93.2	40.5	54.2	47.6	20.1
100	97.4	117.6	36.1	110.3	92.5	40.8	62.6	53.4	23.2
110	91.7	115.6	33.9	108.5	90.7	40.2	70.0	58.5	25.9
120	88.1	114.3	32.6	106.0	88.6	39.2	76.2	62.8	28.2
130	85.5	112.9	31.6	103.4	86.8	38.3	81.3	66.5	30.1
140	83.5	111.8	30.9	101.1	85.4	37.4	85.6	69.4	31.7
150	81.8	110.7	30.3	99.3	84.4	36.8	89.1	71.6	33.0
160	80.4	109.7	29.8	97.7	83.6	36.1	92.1	73.6	34.1
170	79.3	108.9	29.4	96.5	83.0	35.7	94.4	75.1	34.9
180	78.3	108.2	29.0	95.6	82.4	35.4	96.3	76.5	35.6

CP: Calcified plaque; HU: Hounsfield unit; LAP: Low-attenuation non-calcified plaque; NCP: Non-calcified plaque; SD: Standard deviation; TPV: Total plaque volume on T3D images
